# New pockets in dengue virus 2 surface identified by molecular dynamics simulation

**DOI:** 10.1007/s00894-012-1687-6

**Published:** 2012-11-30

**Authors:** Carlos A. Fuzo, Léo Degrève

**Affiliations:** Grupo de Simulação Molecular, Departamento de Química, Faculdade de Filosofia Ciências e Letras de Ribeirão Preto, Universidade de São Paulo, Av. Bandeirantes, 3900, 14040-901 Ribeirão Preto, SP Brazil

**Keywords:** Dengue disease, Envelope protein, Fusion process, Molecular dynamics, Protein pockets

## Abstract

One of the factors limiting the search of new compounds based on the structure of target proteins involved in diseases is the limited amount of target structural information. Great advances in the search for lead compounds could be achieved to find new cavities in protein structures that are generated using well established computational chemistry tools. In the case of dengue, the discovery of pockets in the crystallographic structure of the E protein has contributed to the search for lead compounds aimed at interfering in conformational transitions involved in the pH-dependent fusion process. This is a complex mechanism triggered by the acid pH of the endosomes that leads to the initial changes in the E protein assembly at the virus surface. In the present work, an arrangement of three ectodomain portions of the E protein present on the surface of the mature dengue virus was studied through long all-atom molecular dynamics simulations with explicit solvent. In order to identify new pockets and to evaluate the influence of the acid pH on these pockets, the physiological neutral pH conditions and the acid pH of the endosomes that trigger the fusion process were modeled. Several pockets presenting pH-dependent characteristics were found in the contact regions between the chains. Pockets at the protein-protein interfaces induced by a monomer in another monomer were also found. Some of the pockets are good candidates for the design of lead compounds that could interfere in the rearrangements in E proteins along the fusion process contributing to the development of specific inhibitors of the dengue disease.

**Electronic supplementary material:**

The online version of this article (doi:10.1007/s00894-012-1687-6) contains supplementary material, which is available to authorized users.

## Introduction

Dengue, a viral disease, is a major public health problem affecting more than 100 million people in tropical and subtropical areas annually [1]. It is caused by four serotypes of the dengue virus (DENV), which pertains to the *Flavivirus* genus. This is an enveloped virus of ∼500 Å diameter with an external shell consisting of 180 copies of the envelope (E) protein arranged as 90 homodimers over the viral surface in a herringbone pattern, so-called protein rafts [2–4]. Flaviviruses infect the host cell through a receptor-mediated endocytosis followed by the fusion between the endosome and virus membranes [5–7]. The central event in the infection is the fusion that is a process mediated by E protein rearrangements due to the decrease of pH from neutral to acid in the endosomes [3, 4, 8–12]. It was hypothesized that the E protein rearrangement is triggered by the histidine residues that change their ionization state from uncharged to positively charged in the low-pH environment of the endosome [13–17].

Many efforts to develop antivirals that act on the virus entry stage into the host cell are focused on the E protein [3]. A hydrophobic pocket observed in the crystal structure of E protein of DENV 2 (DENV-2), which is occupied by a detergent molecule, *n*-octyl-*β*-D-glucoside (*β*-OG), has been a primary potential binding site for small molecules that may inhibit the conformational changes in the E protein necessary to the fusion process [18]. Several studies that focused on this pocket found candidates for drugs by searching compound libraries through virtual screening [9–11]. Targeting another pocket in the crystal structure of DENV-2 E protein, located using a cavity-detection algorithm in virtual screening, has also identified an inhibitor of DENV-2 [12]. The knowledge or the detection of the pockets was the main factor that led to finding new drug candidates against dengue. The use of the E protein as a target for antiviral therapy creates the possibility of acting on several routes through the identification of ligand-binding pockets such as those in the transition structures that occur in the E protein during the lifecycle of the virus, in the fusion-active trimer, and in E protein rafts [3]. The E protein rafts densely populate the surface of the mature virus and are interesting starting points for the exploration of the cavities for the search of compounds able to interfere in the E protein rearrangements that lead to the fusion process [3]. Inserted ligands in some of these cavities may act as allosteric modulators of the protein-protein interactions [19] present at the ordered surface of the mature virus. These questions reveal new possibilities, because pockets can be found not only in protein crystals, but also in computer modeled structures such as those found in intermediate states of the E protein, computed using metadynamics [20], and in the structures obtained in molecular dynamics (MD) simulation studies. The latter is extremely interesting, since MD simulation methods are powerful and widely used techniques in understanding the dynamics and structures of proteins [21, 22] since they are able to investigate the protein structures at the atomic level.

MD simulation has been used mainly to study the influence of the histidine protonation on the E protein structure of DENV [13, 14, 20, 23, 24]. In these studies, the effects of the pH changes before and after the internalization of the virus in the endosome were modeled by studying the E protein in two extreme situations, considering that the side chains of all the histidine residues were unprotonated or protonated, and modeling something like the pre-fusion conditions and the acid conditions of the endosomes. Together with the structural protein behavior information, the MD simulations can also be helpful in the discovery of new pockets, through the exploration of protein trajectory snapshots along the simulation time. A large number of algorithms are available for predicting cavities on the surface of proteins [25]. However, the majority of the detection algorithms are only employed for evaluating the pockets in static protein structures, and few computational tools have been developed for the identification of pockets and of their properties along the MD trajectory snapshots. Eyrisch and Helms [26] deal with this problem of successfully detecting transient pockets on the surfaces of the proteins BCL-XL, IL-2, and MDM2 with a proposed protocol for pocket detection with the application of the PASS algorithm [27]. They also demonstrate the improved influence of the solvent polarity and of the backbone movements in the diversity and volume of the pockets by employing this same protocol [28]. In another recent study, Schmidtke et al. [29] have identified by means of their pocket detection algorithm in MD snapshots, named *mdpocket*, open/closed conformations of the pockets along the trajectory. These studies reveal the dynamic behavior of the protein pockets; this behavior is important in the modern design of drugs, since the consideration of multiple conformations of the pockets has been shown to improve the predictive power of docking over crystal structures [30–32].

**Fig. 1:**
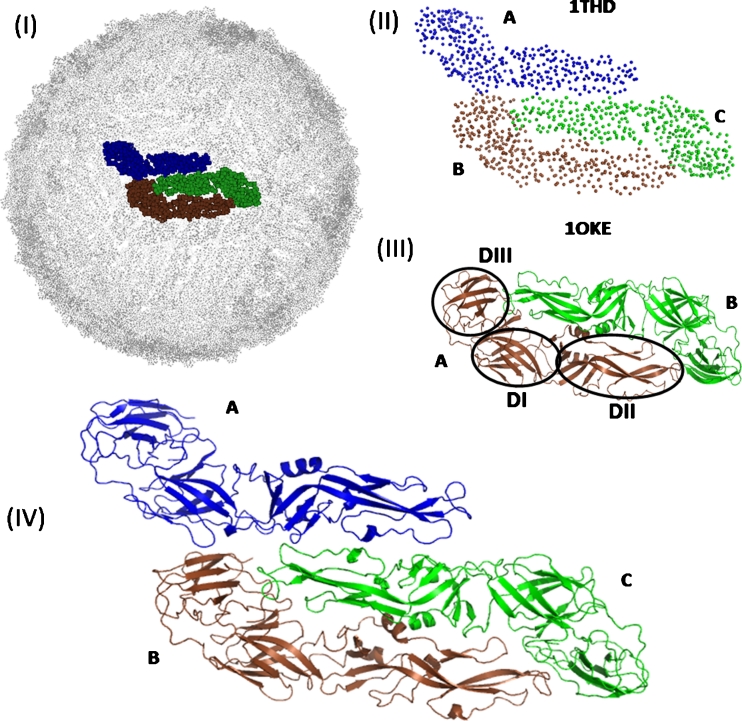
**I** Biological assembly and **II** PDB structure of 1THD containing the C_α_ atoms [33]. **III** All-atom dimer structure [18]. The domains DI, DII, and DIII of E protein are shown within the circles in the A chain. DI, comprising the residue sequences Met1-Asn52, Glu133-Phe193, and Gly281-Gly296; DII is the sequences Pro53-Pro132 and Asn194-Thr280; and DIII is the sequence Met297-LYS394. **IV** Energy minimized T structure constructed from 1THD and 1OKE (T). In all panels, the A, B, and C chains can be distinguished using different colors

In this work, we examine by MD simulation the pockets and their stability on E protein arrangements of three soluble portions of DENV E protein (T), as modeled on the virion surface of mature DENV-2, with atomic details from an all-atom structure constructed from a low resolution structure [33] (Fig. 1). This trimer arrangement, which is a dimer together with a monomer, is one-half of an E protein raft formed by three parallel dimers repeated 30 times along the virus surface, forming an icosahedral fold [2, 4]. Our main objective is the identification of new pockets in a piece of the virus surface and the study of the temporal evolution of these pockets in order to obtain subsidies for the search of compounds that can interfere in the reorganization of the virus surface that leads to the fusion process. With this purpose, we will first study this raft at physiological pH conditions, modeling something like the pre-fusion conditions. In order to map the pockets that are modulated by the acid pH, a study of the same system was carried out by modeling the acid conditions of the endosomes [13–17].

## Models and methods

The monomer structure of the DENV E protein contains 394 amino acid residues spatially distributed in three domains, namely DI, DII, and DIII (Fig. 1III). The C_α_ structure of the DENV-2 E protein, deposited in the Protein Data Bank (PDB) [34] with identification code 1THD [33], was used as a template for the construction of the T initial structure for the simulation studies. The 1THD structure of the trimer containing the A, B and C chains was originally constructed by the superimposition of an all-atom monomer structure (PDB code 1TG8 [33]) onto the mature DENV-2 Cryo-EM density map, considering individually the agglomerates DI/DIII and DII as rigid bodies, resulting in a difference of 0.29 nm in the C_α_ root-mean-square deviation (RMSD) between the monomers of 1THD and 1TG8. However, the monomers of the dimer DENV-2 E protein structure, under the PDB code 1OKE [18], present smaller RMSDs, 0.25 nm and 0.26 nm for the A and B chains of 1THD. The smaller RMSD and the advantage of the good intermonomer contacts in 1OKE are advantages taken into account in the T structure construction, by means of the better C_α_ superposition of 1OKE on the dimer formed by the B and C chains of 1THD and by the best superposition of the A chain of 1OKE on the A chain of 1THD, as shown in Fig. 1.

Two target systems of interest were studied by MD simulation in the present work in order to mimic the physiological pre-fusion condition and the acid condition of the endosome. These conditions were modeled as in previous simulation studies of E protein [13, 14, 20, 23, 24], considering the side chains of histidine residues respectively singly and doubly protonated. In the first system (namely T^0^), the histidine residue side chains were protonated only in position δ or ε, with the aid of the MolProbity [35]. In the second system, the histidine residues were fully protonated in both the δ and ε positions (T^+^). The objective of this strategy, also used in simulations of the dimer of the E protein [13, 14, 23], was to observe the differences in the behavior of the T in different histidine residue protonation states, due to their important role during the viral infection [8]. The two systems were constructed by inserting T^0^ or T^+^ at the center of the parallel piped boxes of sides 21.0, 12.0, and 12.0 nm. The systems were completed using about 99,000 water molecules and Na^+^ and Cl^-^ ions to neutralize the net charges of the proteins and to reach the ionic strength of 150 mM.

The simulations were carried out using the GROMACS 4.5.1 simulation package [36]. The united atom GROMOS96 43A1 force field [37] with the SPC/E model for water was used. Protein covalent bonds involving hydrogen atoms were constrained by the LINCS algorithm [38], while the SETTLE algorithm [39] was used to maintain the rigid structure of the water molecules. The temperature and pressure, regulated by the Berendsen’s algorithms [40], was at 300 K and 1 atm, respectively. A minimal invasive thermostat was applied to control the temperature [41]. A cutoff in the interactions was applied at 1.0 nm, and the particle mesh Ewald summation method [42] was employed to calculate the long-range electrostatic interactions. The simulations were initiated through an energy minimization phase using a steepest descent algorithm, in order to eliminate bad contacts and undesirable forces. After this, for the equilibration of each system, six simulations of 10 ps each were realized by increasing the temperature during the sequence to 50, 100, 150, 200, 250, and 300 K. Finally, the simulations of the two systems were carried out at 500 ns. The MD motion equations were integrated through the *leap-frog* algorithm [43] using 2 fs time steps. The coordinates of all the atoms were recorded every 5 ps for further analysis, which was carried out with the GROMACS tools [44]. The PyMol package [45] was used for the protein structure visualization.

The protein pockets were detected using the *fpocket* and *mdpocket* programs [29], which are based on the geometric α-sphere theory [46], with default parameters employed in order to detect small molecule binding sites, a minimum of 30 α-spheres per pocket with radius between 0.3 and 0.6 nm, clustered consecutively with the three following parameters: (i) the maximum distance between Voronoi vertices of 0.173 nm, (ii) the maximum distance between the centroids of two clusters equal to 0.45 nm, and (iii) the maximum distance between two alpha sphere centers for the multiple linkage clustering steps of 0.25 nm.

## Results and discussion

**Fig. 2:**
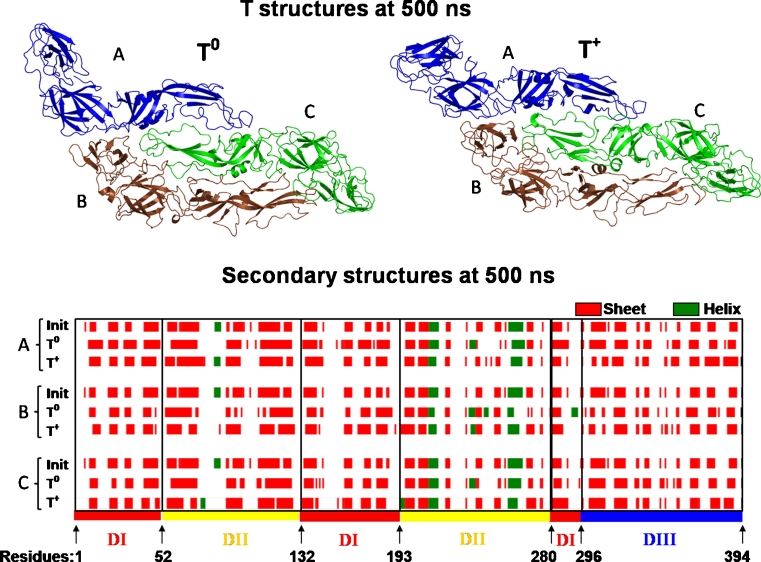
Final structures of T^0^ and T^+^ and the sheet and helix secondary structure elements for the A, B, and C chains in initial and final structures of simulations T^0^ and T^+^

The initial structures of the T were maintained in the computational experiments in the two conditions of the histidine residues protonation, with only some rearrangements in the secondary structures. The arrangements of the monomers in the quaternary structures of the T were maintained up to 500 ns in simulations T^0^ and T^+^ (Fig. 2). The secondary structures of the monomers, determined in the initial and final structures using main chain hydrogen bond (HB) energy criteria [47], revealed that the sheet and helical contents of T^0^ and T^+^ suffer few modifications (Fig. 2) observed in the positions, sizes, and residue contents for almost all of the sheets in relation to the experimental structure and between T^0^ and T^+^. However, these modifications were due to the rearrangement of local structures, without the loss of the general sheet contents. It can also be noted that the two helical regions present in the crystal structure were conserved in DII for all chains in T^0^ and T^+^, while a small helical region in an unstructured segment of DII, with 14 residues (from 74 to 88), was observed only in the A and C chains of T^+^. Other small helical regions appear in T^0^ and T^+^ in initially unstructured regions (Fig. 2) that can be observed mainly in T^0^, where the helical contents appear in the DII region of residues 230 to 250 in all chains, and in the DI of the B chain near residue 294. A new helix was formed near the residue Phe193 of the C chain in T^+^. In a general way, the arrangement and the structures of the T^0^ and T^+^ chains did not suffer major changes when the constructed model used in the beginning of the simulations was immersed in a medium of water molecules and ions in order to model the imposed conditions of physiological ionic strength. However, the results indicate that the protonation of the histidine residues leads to different behaviors in the organization of the secondary structures.

**Fig. 3:**
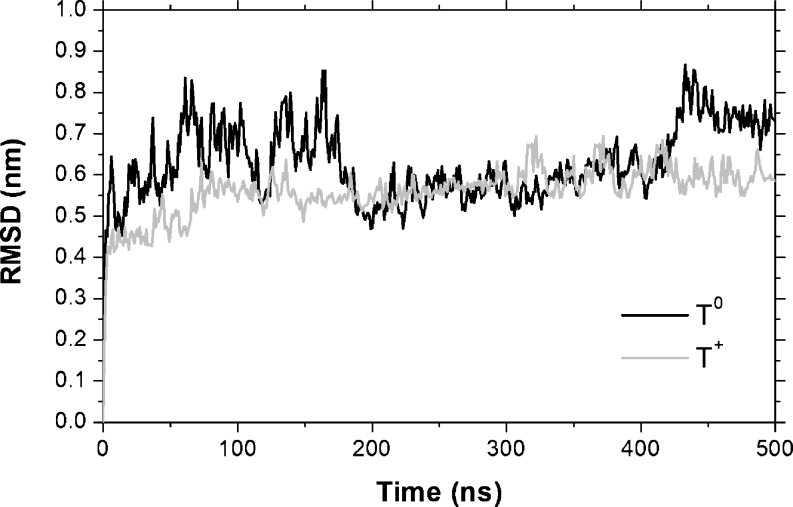
RMSD of T^0^ and T^+^ along the simulations in relation to the initial structure

**Fig. 4:**
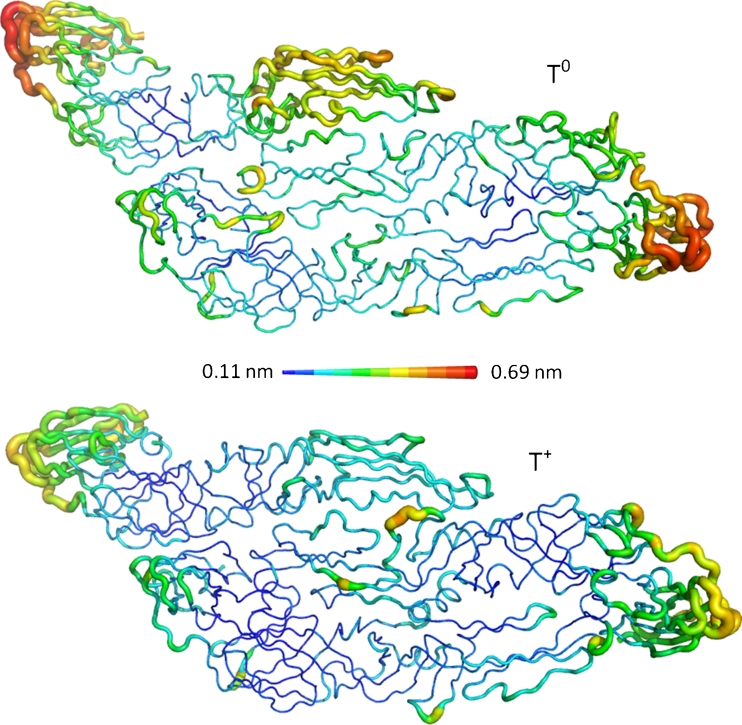
RMSD_res_ in T^0^ and T^+^ simulations calculated from 80 to 500 ns. The values are depicted in the average structure in which the size of the radius of the chain corresponds to RMSD_res_

The behavior of the protein structures throughout the simulations was quantitatively monitored by the RMSD relative to the initial structure, with the purpose of verifying the stability of the T^0^ and T^+^ structures and their behaviors relative to the initial structure (Fig. 3). The complete protonation of the histidine residues resulted in the major stability of the T^+^ structure. The most pronounced deviations relative to the initial structure were observed in the T^0^ simulation, because the RMSD reached values of 0.87 nm, while in T^+^, the largest RMSD value was 0.70 nm. The average RMSD for T^0^ was 0.63 ± 0.09 nm, while for T^+^, it was 0.56 ± 0.06 nm, thereby showing that the chain fluctuations are not statistically different in T^0^ and T^+^. The fluctuations observed in the T^0^ RMSD are consequences of the major movements in the DII and DIII of the A chain and, in domain DIII, of the C chain, where the RMSDs per residue (RMSD_res_), calculated along all the trajectories (Fig. 4), reached values of 0.69 and 0.58 nm for the systems T^0^ and T^+^, respectively. The RMSD_res_ in the DIII of the B chains was smaller, with a maximum value of 0.42 nm, than those observed in the A and C chains in both T^0^ and T^+^ systems. It was almost certainly a consequence of the proximity of the A and C chains that contributed to the stabilization of this region. It can be observed that the contact regions between the chains were the most stable parts of the T in both conditions. As a consequence, the stable regions, modulated by the contacts between the chains, were the regions where the presence of possible ligand-binding sites for the inhibition of virus entry in the host cell can appear, which was seen through the detection of several pockets in these regions.

**Fig. 5:**
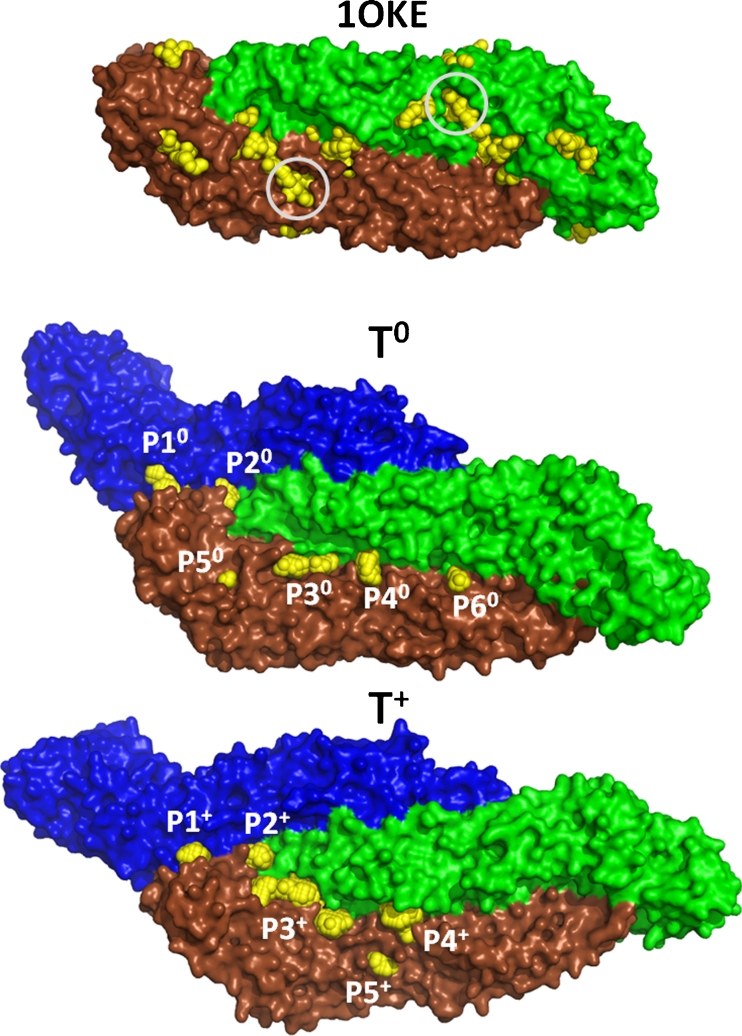
Pockets (*yellow* spheres) detected in the surface of 1OKE and in the frames from 80 to 500 ns in the T^0^ and T^+^ simulations. The circles in the 1OKE representation indicate the position of the *β*-OG binding sites in both chains. The T^0^ and T^+^ structures were obtained by averaging the atomic positions along the frames used for pocket detection

The presence of pockets was first tested on the experimental structure of the DENV-2 E protein (1OKE) dimer, with the objective of verifying whether the methodology was able to find the binding site where the *β*-OG detergent is located, at the junction of the DI and DII domains of both monomers [18]. The sugar molecules, the *β*-OG detergent, and the water molecules were removed from the 1OKE structure, allowing an investigation of the protein pockets without interferences. The method was able to find the binding site of the *β*-OG in each monomer as well as finding other pockets (Fig. 5I), including a pocket at the junction of DI and DIII that was a target in previous inhibitor binding studies [12].

The existence and properties of the pockets in the T^0^ and T^+^ trajectories were investigated by applying the *mdpocket* in the structures produced from 80 to 500 ns with a time step of 0.5 ns in order to identify pockets that appear in at least 20 % of the frames. The first 80 ns were cut out because they are relaxation runs. The calculations were applied to the trajectory where the C_α_ atoms of all chains were fitted on the averaged structure obtained between 80 and 500 ns, according to the use of the grid-based method for the pocket identification. The pocket grids obtained are shown as yellow spheres in Fig. 5II and III for T^0^ and T^+^, respectively. Six pockets were found in T^0^ (namely from P1^0^ to P6^0^) and five pockets in T^+^ (P1^+^ to P5^+^). Four identically localized pockets were identified in T^0^ and T^+^, located mainly in the stable regions observed by the RMSD_res_ data (Fig. 4); they are P1^0^ and P1^+^, P2^0^ and P2^+^, P3^0^ and P3^+^, and P4^0^ and P4^+.^. This feature indicates that, on the surface of the virus where the chains forming the capsid are largely organized, the contact between the E protein monomer stabilizes the structures, leading to the formation of several pockets (Fig. 1). Another observation is that the cavity corresponding to the binding site of the *β*-OG was found only in the B chain of T^+^ (pocket P5^+^). The nonoccurrence of this pocket in the other chains is not surprising, since this pocket was induced by the presence of the detergent obviously missing in the protein crystals grown in the absence of the detergent [18]. The reproducibility of the data is confirmed by the detection of the corresponding pockets of T^+^ in a set of three additional simulations at different ionic strengths (0, 75, and 225 mM) carried out for 250 ns (Supplementary Fig. S1).

**Fig. 6:**
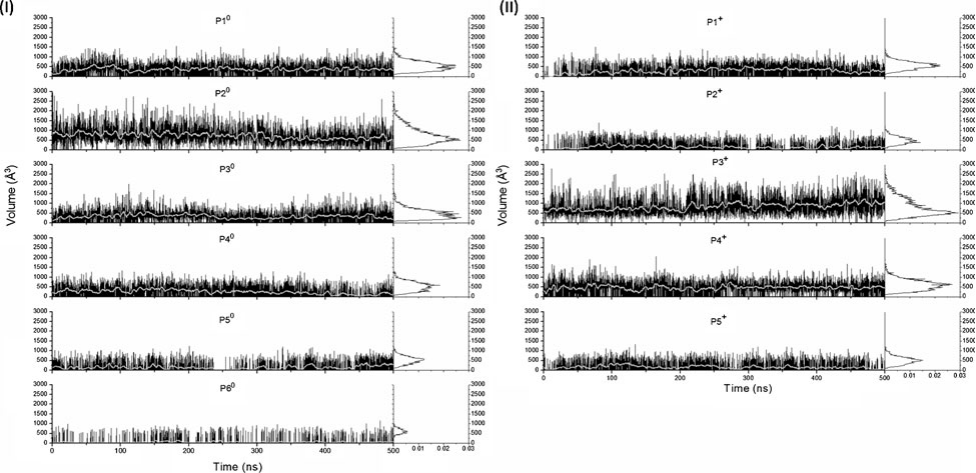
The volumes of the pockets along the time for T^0^ (**I**) and T^+^ (**II**) obtained every 0.1 ns (*black* lines) and the smoothed volumes (*gray* lines). The histograms of the volumes on the right are normalized by the number of points employed in the volume calculation. The volumes during the first 80 ns period, considered as the relaxation phase and excluded from the *mdpocket* analysis, were included in the figure in order to show the entire evolution of the pockets along the whole simulation

A list of the residues was constructed for each detected pocket when at least one of its atoms was distant from the center of a pocket’s sphere by a maximum of 0.6 nm (Table S1). These lists were employed for the screening of the respective pockets gathered by the *fpocket* algorithm to individual frames that identified the neighboring residues of each pocket by the same distance criteria used for the list construction. The fluctuations of the protein structure can lead to changes in the composition of the residues of the pockets. For this reason, the presence of at least 50 % of its residues were used as the criterion for identification of the targeted pocket. The volume of the pockets throughout the time and their distribution was extracted from data collected at every 0.1 ns in the systems T^0^ and T^+^, and it is depicted in Fig. 6I and II, respectively. In both T^0^ and T^+^ systems, the volume of the pockets oscillates along the simulation time, revealing the dynamic behavior of the pockets dimension that present opening and closing behaviors. Throughout most of the analyzed trajectories, zero pocket volumes were also observed, when the respective pocket was not detected, leading to a pocket occurrence fraction ranging from 0.26 for P5^0^, to 0.99 for P3^0^ (Table 1). The comparison of the characteristics of the T^0^ and T^+^ four equivalent pockets shows that only small differences can be observed in the volumes and in the occurrence fractions comparing P1^0^ and P1^+^. On the other hand, a large decrease of the mean volume and of the occurrence fraction of P2^+^ is observed when compared with P2^0^. Increases are perceived of the mean volumes of the P3^+^ and P4^+^ by factors of 2.56 and 1.97, respectively, compared to the T^0^ results. Interestingly, a large increase in the extension of the pocket P3^+^ is also noted that results in a practically complete occupation of the whole junction between the DI of the B chain and the DII of the C chain. The pockets equivalent to the P5^0^ and P6^0^ are not found in T^+^. The pocket P5^0^, located at the junction between the DI and DIII of the B chain, is located in the same region of the pocket found in the 1OKE experimental structure that had been the target of previous inhibitor binding studies [12].

**Table 1:** Average volumes and fraction of time in which the volumes are greater than zero of the detected pockets calculated in the intervals from 80 to 500 ns for T^0^ and T^+^

	T^0^			T^+^	
Pocket	Volume (Å^3^)	Fraction	Pocket	Volume (Å^3^)	Fraction
P1^0^	368 ± 314	0.68	P1^+^	305 ± 319	0.53
P2^0^	701 ± 415	0.95	P2^+^	144 ± 246	0.29
P3^0^	333 ± 304	0.68	P3^+^	854 ± 452	0.99
P4^0^	239 ± 308	0.44	P4^+^	470 ± 334	0.77
P5^0^	140 ± 256	0.26	P5^+^	163 ± 262	0.32
P6^0^	49 ± 172	0.68	−	−	−

The histidine residues are not fully protonated, found before the virus infection, so that targeting the pockets found in T^0^ can be an important approach to discovering new drugs that can interfere in the formation of the active virus surface, in this way inhibiting the reorganization of the E proteins triggered by the endosome acid pH. The P1^0^, P2^0^, P3^0^, P4^0^, and P6^0^ pockets are located in the stable regions of the contact between the chains, that is, they were located in the regions that stabilized the overall structure of T. In this sense, ligands of the P1^0^ pocket can stabilize the contact between the A and B chains. Furthermore, the P2^0^ pocket is interesting because it is located exactly in the region, firstly, where the three chains are in contact, and secondly, it is extremely stable along all the times. The other pockets, P3^0^, P4^0^, and P6^0^, are located between the B and C chains so that they can also be relevant targets for increasing the stabilization of the dimer form of the E protein. The region of the P3^0^ pocket shows an interesting feature, since the volume average increases from 333 Å^3^ for P3^0^ to 854 Å^3^ for P3^+^. This opening is probably related to an initial step in the destabilization of the dimer structure induced by the complete protonation of the histidine residues. This characteristic has also been observed in other MD studies of the dimer [13], where the histidine residues are single or doubly protonated, modeling the pHs of 7 and 6, respectively. This feature allows identification of the region of the pockets P3^0^ and P3^+^ as preferential targets for the design of compounds interfering in the dimer of the E protein rearrangements that can lead to the fusion. A recent study has shown that after the cleavage of the prM protein, the proteolytic product pr remains associated to the DENV blocking the membrane fusion at acid pH [48]. Interestingly, this activity inhibition is attributed to the dimer stabilization because the pr makes contact with both monomers [48] in the same region where the pockets P3^0^ and P3^+^ were found. Another interesting pocket is P5^0^, since it exists solely in the pre-fusion modeled conditions and in the crystallographic E protein structure. Despite this pocket’s existence only in the B chain, it may play a role in the stabilization of the mature virus surface by allosteric modulation. The use of the pocket present in the region of P5^0^ of the crystallographic structure of DENV-2 as target has identified an inhibitor of DENV [12]. The pockets modulated by the acid pH are interesting for the design of inhibitors due to the pH-dependent fusion mechanism of E protein. The use of the conformation sampling obtained by the MD simulation offers more realistic descriptions of the pocket behaviors, improving the search of ligands that can be candidates to be tested as inhibitors. However, a challenging problem is to perform, with compounds listed in large databases, the search of these compounds in pockets that exhibit dynamically changing structures. This fact increases the amount of calculations and information in virtual screening studies. Exploratory screenings, like the ones concluded in recent studies [30], have to be applied to identify the best structures of the pockets along the trajectories in future virtual screening studies.

Applying the same protocol and considering the chains individually, pockets occur at the interface between the B and C chains of T^0^ and only in the B chain of T^+^ (Fig. S2). These pockets, which are also found in the crystallographic 1OKE structure, are located along the interface between DI and DIII within the same chain. In the case of the B chain of T^0^, the N-terminal loop separates the large pocket observed in the C chain of T^0^ into two smaller pockets. The absence of the pockets in the A chain at the same region of the detected pockets at the B and C chains indicates that they are induced by the other monomer. These pockets are mainly occupied by the residues Trp101 and Phe108 that are part of the fusion peptide, which is responsible for the attachment of the E protein to the host membrane in the fusion process [6]. In contrast with the pockets detected early in modeled virus surfaces under neutral pH, these cavities can be targets for the study of compounds that could act on steps such as the transition for the formation of the active trimer structure, once these pockets are located in the contact region of DIII with DI in the post-fusion structure [6]. Another route can be to focalize the formation of the mature virus, since ligands of these pockets can interfere with the organization of the dimers that constitute the surface of the mature virus.

## Conclusions

The present study describes the behavior of the all-atom T structure of the dengue virus E protein through explicit solvent MD simulation. Long-duration simulation studies are quite important because they enable a better understanding of the focused protein’s structure by means of the detailed features of its structures. The analysis of the MD results shows that the built structures are stable, particularly the contact regions between the chains, which are very stable. The pockets detected in these regions must be the subject of future research for ligands by virtual screening methods. The results of the simulations of the T with single and fully protonated histidine residue side chains show that some pockets appear to be dependent on histidine residue protonation, that is, they depend on the pH. This information helps to select E protein pockets that are susceptible to structural pH-dependent rearrangements. The three pockets that are natural candidates for these searches are P2^0^, P3^0^ and P5^0^. These results contribute to the opening up of new approaches toward the study of inhibitors of the dengue disease processes, through identification of pockets in the virus surface that must be likely targets of compounds to interfere in the E protein rearrangements that lead to fusion processes.

## Electronic supplementary material

Below is the link to the electronic supplementary material.

Table S1 Lists of neighboring residues of the pockets detected in T^0^ and T^+^. These lists were used in the volume calculations of the individual frame pockets employing *fpocket. (DOC 32 kb)*

Fig. S1 Pockets (yellow spheres) detected in the surface of simulations of T^+^ at varied ionic strength shown in order to verify the reproducibility of the simulations. (DOC 1382 kb)

Fig. S2 (I) Pockets (yellow spheres) detected at the interface between the B and C chains of T^0^ and for B chain of T^+^. (II) Volume of the respective pockets shown in (I) calculated along the time every 0.1 ns (black lines) and the smoothed volumes (gray lines). The histograms of the volumes normalized by the number of points employed in volume calculation are also included. (DOC 523 kb)
