# Supplementary material for: New pockets in dengue virus 2 surface identified by molecular dynamics simulation
Source: J Mol Model. 2012 Nov 30;19(3):1369–77. doi: 10.1007/s00894-012-1687-6 (PMC3578724; doi:10.1007/s00894-012-1687-6)
Supplement: Supplementary file 1 — Lists of neighboring residues of the pockets detected in T0 and T+. These lists were used in the volume calculations of the individual frame pockets employing fpocket. (DOC 32 kb) [file 894_2012_1687_MOESM1_ESM.doc]

**Table S1** Lists of neighboring residues of the pockets detected in T^0^ and T^+^. These lists were used in the volume calculations of the individual frame pockets employing *fpocket.*

| **Pocket** | **Chain** | | |
| --- | --- | --- | --- |
|  | **A** | **B** | **C** |
| P1^0^ | 47, 49-51, 131, 134-138, 163-166 | 308, 311, 386-390 | - |
| P2^0^ | 51-54, 128-134, 188, 192-197, 210 | 310-314, 320, 389-393 | 76, 106-108 |
| P3^0^ | - | 2, 44, 46, 153-155, 266-270, 272, 278-279 | 239-251 |
| P4^0^ | - | 61, 125, 204, 206, 257-258, 260-262, 269 | 63-65, 237, 239, 251-256 |
| P5^0^ | - | 1-2, 140-143, 149-152, 156-160, 176 | - |
| P6^0^ | - | 237-239, 249-254 | 203-204, 272-273 |
| P1^+^ | 133-134, 136, 165, 167-170 | 305-309, 325, 327-328, 387 | - |
| P2^+^ | 132-133 | 309-312 | 74-77, 101, 104-108 |
| P3^+^ | - | 2-3, 6-7, 26-30, 43-48, 138-140, 147-158, 161, 276-278, 309-312, 321-323, 364-366 | 72-74, 97-107, 111-113, 241, 243, 246-249 |
| P4^+^ | - | 62, 122-124, 201-204, 255, 272-274 | 62-65, 122-123, 250-255 |
| P5^+^ | - | 49-52, 126, 128, 136, 200, 202, 271, 274-276 | - |
