# Supplementary material for: New pockets in dengue virus 2 surface identified by molecular dynamics simulation
Source: J Mol Model. 2012 Nov 30;19(3):1369–77. doi: 10.1007/s00894-012-1687-6 (PMC3578724; doi:10.1007/s00894-012-1687-6)
Supplement: Supplementary file 2 — Pockets (yellow spheres) detected in the surface of simulations of T+ at varied ionic strength shown in order to verify the reproducibility of the simulations. (DOC 1382 kb) [file 894_2012_1687_MOESM2_ESM.doc]

**
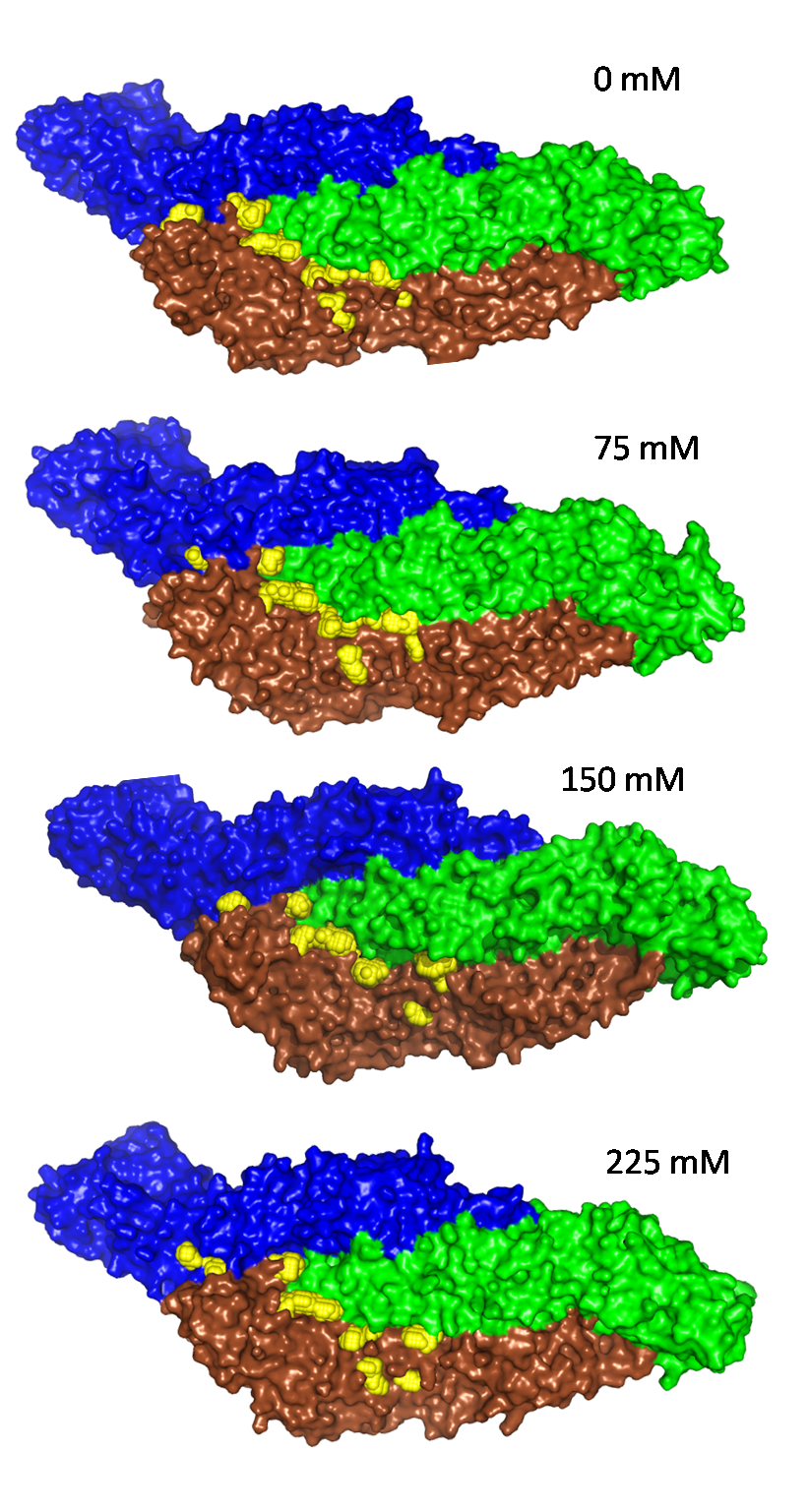
**

**Fig. S1** Pockets (yellow spheres) detected in the surface of simulations of T^+^ at varied ionic strength shown in order to verify the reproducibility of the simulations.
