# Supplementary material for: New pockets in dengue virus 2 surface identified by molecular dynamics simulation
Source: J Mol Model. 2012 Nov 30;19(3):1369–77. doi: 10.1007/s00894-012-1687-6 (PMC3578724; doi:10.1007/s00894-012-1687-6)
Supplement: Supplementary file 3 — (I) Pockets (yellow spheres) detected at the interface between the B and C chains of T0 and for B chain of T+. (II) Volume of the respective pockets shown in (I) calculated along the time every 0.1 ns (black lines) and the smoothed volumes (gray lines). The histograms of the volumes normalized by the number of points employed in volume calculation are also included. (DOC 523 kb) [file 894_2012_1687_MOESM3_ESM.doc]

**
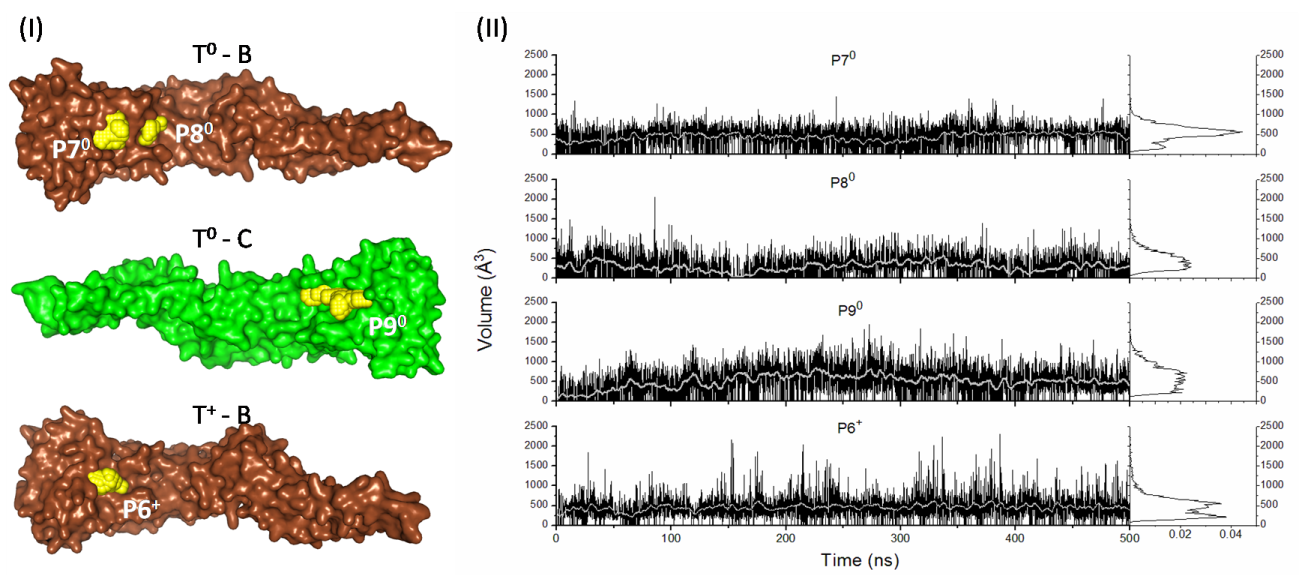
**

**Fig. S2** (I) Pockets (yellow spheres) detected at the interface between the B and C chains of T^0^ and for B chain of T^+^. (II) Volume of the respective pockets shown in (I) calculated along the time every 0.1 ns (black lines) and the smoothed volumes (gray lines). The histograms of the volumes normalized by the number of points employed in volume calculation are also included.
